# Optical coherence tomography—in situ and high-speed 3D imaging for laser materials processing

**DOI:** 10.1038/s41377-022-00981-8

**Published:** 2022-09-23

**Authors:** Xiaodong Wang, Xin Yuan, Liping Shi

**Affiliations:** 1 School of Engineering, Westlake University, Hangzhou, 310030 Zhejiang China; 2 Key Laboratory of 3D Micro/Nano Fabrication and Characterization of Zhejiang Province, Westlake University, 18 Shilongshan Road, Hangzhou, 310024 Zhejiang China; 3 Institute of Advanced Technology, Westlake Institute for Advanced Study, 18 Shilongshan Road, Hangzhou, 310024 Zhejiang Province China; 4 Wuhan National Laboratory for Optoelectronics, Huazhong University of Science and Technology, 1037 Luoyu road, Wuhan, 430079 China

**Keywords:** Imaging and sensing, Laser material processing

## Abstract

Exploiting Fourier-domain optical coherence tomography, a high-speed and real-time inspection of multi-photon 3D laser printing has been reported. We expect that this technique can be further extended to single shot compressive 3D imaging for studying the ultrafast dynamics of laser-matter interaction.

Femtosecond lasers are essential tools for micro/nanoscale surface patterning^1^, as well as 3D lithography and printing^2,3^. Aiming for highly precision laser engineering, in situ and high-speed monitoring is of significance. While the wide-field microscopy enables a convenient in situ monitoring, it is limited to 2D imaging. The other advanced imaging methods, such as scanning electron microscopy, holographic tomography^4^ and X-ray tomography^5^, are capable of capturing 3D images. However, they need to be conducted in an ex-situ manner, which cannot provide real-time monitoring of fabrication defects and may result in irreversible damages to the fabricated structures. Although coherent anti-Stokes Raman scattering (CARS) microscopy^6^**—**measuring the spectrally filtered CARS signal of the targeted samples**—**provides an in situ 3D imaging, it suffers a relatively low imaging speed. Therefore, it remains a big challenge to achieve noninvasive in situ and high-speed 3D imaging during laser fabrication.

To address this challenge, the work of Zvagelsky et al. now offers a promising solution by utilizing optical coherence tomography (OCT)^7^. The OCT, which has been widely used in ophthalmology, holds promising potential in in situ 3D imaging^8,9^. To be concrete, OCT measures the backscattered light from different layers of a sample to generate 3D reconstruction. Fourier-domain OCT (FD-OCT) provides an efficient way to implement the low-coherence interferometry. Instead of recording interference intensity at different axial planes of the reference arm, FD-OCT measures the spectral intensity as a function of light wavelength. The spectral interference signal can be translated to the reflective signal from different layers of the sample. As a result, 3D structures can reliably be retrieved with a Fourier transform of spectral intensity. FD-OCT supports microscopic resolution, fast imaging speed and high sensitivity, rendering it as an ideal candidate for in situ 3D inspection during multi-photon 3D laser printing.

As schematically illustrated in Fig. 1, FD-OCT utilizes the low coherence of a broadband superluminescent diode (SLD) to measure optical path length delays in a sample. Light emission from a SLD is split into two arms through a fiber coupler (FC). The reference arm transmits through a fiber collimator and reflects back into FC after reflection from reference mirror (RM). The sample arm collects the backscattered light from different depth. The sample beam after collimation is scanned through MEMS mirror and focused into the sample by an immersed objective lens. The backscattered light from different sample layers will interact with the reflected reference light to generate the spectral interference signal, which is further collected by a spectrometer. By performing Fourier transform of the spectral interference data, 3D reconstruction of various structures can be obtained, as shown in the inset of Fig. 1.

**Fig. 1:**
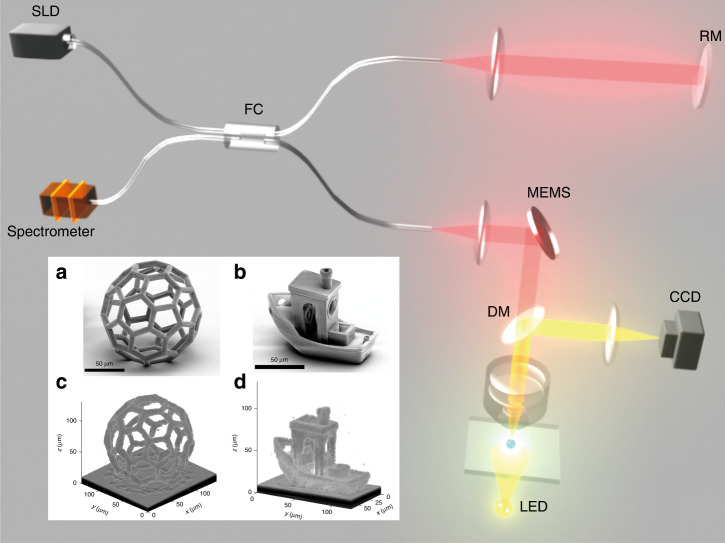
Experimental concept of FD-OCT. Inset: SEM (**a**, **b**) and reconstructed images by FD-OCT (**c**, **d**) of laser-printed 3D structures

Taking advantage of this custom-built OCT system, Zvagelsky et al. have achieved a 1.9 μm lateral resolution, 2.7 μm axial FWHM, up to 1 mm axial field-of-view for one A-scan, and 40 μs acquisition time for one A-scan (equivalent to a frame rate of 25 kHz). On top of this, a wide range of applications were demonstrated to validate the real-life inspections for this OCT instrument, including photoresist-homogeneity inspection before printing, in situ evaluation of the laser-power-dependent refractive index of polymerised regions, time-dependent diffusion processes, thickness measurements of printed parts, surface roughness and volume-homogeneity inspection of printed parts and the comparison between 3D reconstructions of printed microstructures and the targeted 3D computer model.

In conclusion, this work has combined the FD-OCT technology with multi-photon 3D laser printing. A fast and in situ 3D imaging with micrometer resolution has been achieved. We forecast that the advanced OCT-related techniques such as single shot ultrafast 3D imaging^10^, will find more practical applications in investigating the physics of femtosecond laser-material interaction.
